# N-Acetylcysteine Decreases Myocardial Content of Inflammatory Mediators Preventing the Development of Inflammation State and Oxidative Stress in Rats Subjected to a High-Fat Diet

**DOI:** 10.1155/2023/5480199

**Published:** 2023-03-11

**Authors:** Klaudia Sztolsztener, Wiktor Bzdęga, Katarzyna Hodun, Adrian Chabowski

**Affiliations:** Department of Physiology, Medical University of Bialystok, 15-089 Bialystok, Poland

## Abstract

Arachidonic acid (AA) is a key precursor for proinflammatory and anti-inflammatory derivatives that regulate the inflammatory response. The modulation of AA metabolism is a target for searching a therapeutic agent with potent anti-inflammatory action in cardiovascular disorders. Therefore, our study aims to determine the potential preventive impact of N-acetylcysteine (NAC) supplementation on myocardial inflammation and the occurrence of oxidative stress in obesity induced by high-fat feeding. The experiment was conducted for eight weeks on male Wistar rats fed a standard chow or a high-fat diet (HFD) with intragastric NAC supplementation. The Gas-Liquid Chromatography (GLC) method was used to quantify the plasma and myocardial AA levels in the selected lipid fraction. The expression of proteins included in the inflammation pathway was measured by the Western blot technique. The concentrations of arachidonic acid derivatives, cytokines and chemokines, and oxidative stress parameters were determined by the ELISA, colorimetric, and multiplex immunoassay kits. We established that in the left ventricle tissue NAC reduced AA concentration, especially in the phospholipid fraction. NAC administration ameliorated the COX-2 and 5-LOX expression, leading to a decrease in the PGE2 and LTC4 contents, respectively, and augmented the 12/15-LOX expression, increasing the LXA4 content. In obese rats, NAC ameliorated NF-*κ*B expression, inhibiting the secretion of proinflammatory cytokines. NAC also affected the antioxidant levels in HFD rats through an increase in GSH and CAT contents with a simultaneous decrease in the levels of 4-HNE and MDA. We concluded that NAC treatment weakens the NF-*κ*B signaling pathway, limiting the development of myocardial low-grade inflammation, and increasing the antioxidant content that may protect against the development of oxidative stress in rats with obesity induced by an HFD.

## 1. Introduction

N-acetyl-cysteine or N-acetylcysteine (NAC) is a wide-known drug acknowledged by the World Health Organization (WHO) as an essential medication. It is commonly used to treat paracetamol overdose, likewise as a mucolytic agent in certain respiratory diseases [1]. In many countries, it is available as an over-the-counter drug or nutraceutical, known for its antioxidative properties. Firstly, this acetylated precursor of L-cysteine has a direct antioxidant impact due to the free thiol group property—counteracting reactive oxygen species (ROS) and reactive nitrogen species (RNS) as well. Indirectly, by upregulating the intracellular cysteine level NAC increases the rate of reduced glutathione (GSH) synthesis, the most common cellular antioxidant. Additionally, it possesses significant anti-inflammatory properties as a result of nuclear factor *κ* B (NF-*κ*B) suppression followed by diminished production of proinflammatory cytokines, i.e., tumor necrosis factor *α* (TNF-*α*), interleukin 1 (IL-1), and interleukin 6 (IL-6) [1]. Some studies have shown the effect of NAC on the modulation of arachidonic acid (AA) metabolism as the main inflammatory lipid precursor, which belongs to *n* − 6 polyunsaturated fatty acids (PUFA). The effect of NAC on AA metabolism and prostaglandin formation has been demonstrated in activated monocytes and neurons after nerve tissue injury [1, 2]. The administration of NAC together with specific and nonspecific cyclooxygenase (COX) inhibitors—rofecoxib and diclofenac— significantly reduced the formation of prostaglandin E2 (PGE2) induced by lipopolysaccharides, enhancing the action of the above-mentioned COX inhibitors [2].

Given its properties, NAC is intensively studied in various clinical studies covering chronic metabolic disorders, including cardiovascular diseases (CVD), metabolic syndrome, liver complications, and psychiatric illnesses, in which oxidative stress and inflammation are considered as risk factors for the development of mentioned conditions [3, 4]. It is not debatable that the pandemic of obesity and other metabolic syndrome components has a huge impact on chronic cardiovascular diseases, which possess a relevant, growing problem in the global healthcare system [5]. The chronic enhanced availability and influx of fatty acids (FA) favors ectopic lipid accumulation in peripheral tissues such as the liver and kidneys [6–8]. Consequently, an increase in lipid storage intensifies the low-grade inflammation, the impairment in mitochondrial functioning, and oxidative stress development in the cardiac tissue favoring myocardial cell death and heart failure [9, 10]. So, it is important to search for a therapeutic agent that will reveal new potentially anti-inflammatory and antioxidative properties to improve cardiac functioning. Several clinical trials and animal studies have shown that NAC exerts noteworthy actions in cardiovascular disorders, particularly in acute myocardial ischemia and acute myocardial infarction, although its role in chronic cardiovascular diseases is still not fully understood [11, 12]. Thus, our study aims to determine the potential protective impact of N-acetylcysteine supplementation on the occurrence of myocardial inflammation in rats with obesity induced by a high-fat diet (HFD). In this sense, we will examine how NAC can alter the inflammatory response by suppressing the activation of the arachidonic acid pathway. We will also explore the influence of NAC on enzymatic and nonenzymatic antioxidant protection and the products of lipoperoxidation in cardiac injury induced by high-fat feeding.

## 2. Materials and Methods

### 2.1. Animals and Experimental Protocol

The experiment was conducted for eight weeks on male, four-week-old Wistar rats with an initial body weight of approximately 50–70 g. All animals were housed throughout the entire duration of the study in standard laboratory animal living conditions: plastic autoclavable cages, 22 ± 2°C air temperature, a 12 h reverse light/dark cycle, and unlimited access to water and standard rodent chow. After the first week of adaptation to the new environment, the rats were randomly divided into four groups in equal numbers: 10 rats per experimental group; 40 rats for all groups in the experiment. The characteristics of the groups were as follows: (1) control group—rats fed a standard rodent chow (65.5% calories from carbohydrate, 24.2% calories from protein, and 10.3% calories from fat; nutritional composition of the standard diet is presented in the Table 1; and Agropol, Motycz, and Poland); (2) the HFD group—rats fed a high-fat diet (59.8% calories from fat, 20.1% calories from protein, and 20.1% calories from carbohydrate; nutritional composition of the high-fat diet is shown in the Table 2; D12492, Research Diet, New Brunswick, NJ, USA); (3) NAC group—rats fed the above-described standard diet plus N-acetylcysteine (Sigma-Aldrich, St. Louis, MO, USA); and (4) HFD + NAC group—rats fed a high-fat diet as well as N-acetylcysteine. The experimental model of high-fat feeding was selected based on an accessible protocol to contribute to hyperlipidemic occurrence as a crucial factor for the development of obesity-related heart diseases [13–15]. The NAC solution was administered to the appropriate groups once daily, between 8-9 am. The substance was dissolved in a saline solution and immediately administered intragastrically by gastric gavage at a dose of 500 mg/kg of body weight. The individuals from the control and HFD groups received only saline solution. The amount of administered NAC was adjusted according to the current body weight of rats, and it was recalculated every two days. The intragastric administration of NAC ensured that rats obtained the full dose appropriate for body weight. The NAC dose was established based on available data to provide a satisfactory effect and eliminate the risk of toxicity in Wistar rats [16]. The NAC solution was supplemented concomitantly with standard or high-fat diets to determine the potential preventive effect of NAC on cardiac lipid metabolism with particular reference to inflammatory and oxidative alterations. At the end of the eight weeks, after a 12-hour overnight fast, the animals were anesthetized by intraperitoneal phenobarbital injection (80 mg/kg of body weight). The left ventricle was excised and immediately frozen in liquid nitrogen using precooled aluminum thongs. Also, blood was collected in the tubes containing EDTA and then centrifuged to obtain plasma fractions. All samples (left ventricle tissue and plasma) were stored at −80°C until further measurements. The study was approved by the Ethical Committee for Animal Testing in Bialystok (No. 21/2017).

### 2.2. Determination of the Myocardial and Plasma Arachidonic Acid Concentration

Lipids obtained from the left ventricle and plasma samples were extracted using a solution of chloroform/methanol at a 2 : 1 volume ratio according to the procedure previously described by Folch et al. [17]. Then, an internal standard composed of heptadecanoic acid, diheptadecanoic acid, and triheptadecanoic acid was added to the obtained extracts. In the next step, the extracts were distributed on silica gel-coated glass chromatographic plates (Silica Plate 60, 0.25 mm; Merck, Darmstadt, Germany) and separated into four individual lipid fractions—phospholipid (PL), triacylglycerol (TAG), diacylglycerol (DAG), and free fatty acid (FFA)—by the Thin-Layer Chromatography (TLC) method in the separation buffer composed of heptane/isopropyl ether/acetic acid at 60 : 40 : 3 volume ratio. The obtained eluents comprising the individual lipid fractions were trans-methylated in a 14% boron trifluoride-methanol solution and dissolved in hexane. Following that, the Gas-Liquid Chromatography (GLC; Hewlett-Packard 5890 Series II gas chromatograph equipped with a flame ionization detector and Hewlett-Packard-INNOWax capillary column; Agilent Technologies, Santa Clara, CA, USA) method was used to quantify the particular fatty acid methyl esters (FAME) level in each lipid fraction, depending on the retention times of the standard, as previously described by Chabowski et al. [18]. On the basis of the fatty acid composition in the selected lipid fractions, the concentration of arachidonic acid was estimated and expressed in nanomoles per milliliter of plasma or per gram of tissue.

### 2.3. Immunoblotting

The Western blot technique was used to measure the expression of proteins included in eicosanoid synthesis pathways and the inflammatory response.

In brief, before the immunoblotting procedure, samples of the left ventricle tissue were homogenized in a radioimmunoprecipitation assay (RIPA) buffer containing a cocktail of phosphatase and protease inhibitors (Roche Diagnostic, Mannheim, Germany). In prepared tissue homogenates, the total protein concentration was assessed with the bicinchoninic acid (BCA) method using bovine serum albumin (BSA) as a standard. The homogenates were reconstituted in Laemmli buffer (Bio-Rad, Hercules, CA, USA) to obtain the same amount of protein (30 *μ*g) and separated on 10% Criterion TGX Stain-Free Precast Gels (Bio-Rad, Hercules, CA, USA) during electrophoresis. After that, proteins were transferred onto membranes—polyvinylidene fluoride (PVDF) or nitrocellulose membranes, which are suitable for semi-dry or wet conditions, respectively. After blocking in tris-buffered saline complemented with Tween-20 (TBST) with 5% nonfat dry milk or 5% BSA, the membranes were incubated overnight with proper primary antibodies as follows: cyclooxygenase-1 (COX-1, sc-19998, 1 : 500; Santa Cruz Biotechnology, Inc., Dallas, TX, USA), cyclooxygenase-2 (COX-2, sc-166475, 1 : 500; Santa Cruz Biotechnology, Inc., Dallas, TX, USA), 5-lipoxygenase (5-LOX, ab169755, 1 : 1500; Abcam, Cambridge, UK), 12/15-lipoxygenase (12/15-LOX, sc-133085, 1 : 500; Santa Cruz Biotechnology, Inc., Dallas, TX, USA), nuclear factor erythroid 2-related factor 2 (Nrf-2, sc-365949, 1 : 500; Santa Cruz Biotechnology, Inc., Dallas, TX, USA), transforming growth factor *β* (TGF-*β*, 3711, 1 : 500; Cell Signaling Technology, Danvers, MA, USA), nuclear factor *κ* B (NF-*κ*B, 4764, 1 : 500; Cell Signaling Technology, Danvers, MA, USA), and B cell lymphoma 2 (Bcl-2, 2870, 1 : 1000; Cell Signaling Technology, Danvers, MA, USA). In the next stage, the membranes were incubated with appropriate secondary antibodies conjugated with horseradish peroxidase (HRP), i.e., anti-rabbit (7074, 1 : 3000; Cell Signaling Technology, Danvers, MA, USA) and anti-mouse (sc-546102, 1 : 3000; Santa Cruz Biotechnology, Inc., Dallas, TX, USA). Then, the protein bands were visualized using a chemiluminescent substrate (Clarity Western ECL Substrate; Bio-Rad, Hercules, CA, USA), and the obtained signals were densitometrically measured using the ChemiDoc (Image Laboratory Software; Bio-Rad, Warsaw, Poland). The expression of each analyzed protein was normalized to the total protein expression and expressed as the percentage of the control group (100%).

### 2.4. Determination of the Myocardial Arachidonic Acid Derivatives and Oxidative Stress Parameters

We applied the commercial enzyme-linked immunosorbent assay (ELISA) and the colorimetric assay kits to determine the concentration of arachidonic acid derivatives—prostaglandin E2 (Cusabio, Houston, TX, USA), prostaglandin I2 (PGI2; Cusabio, Houston, TX, USA), leukotriene B4 (LTB4; Cusabio, Houston, TX, USA), leukotriene C4 (LTC4; MyBioSource, San Diego, CA, USA), lipoxin A4 (LXA4; MyBioSource, San Diego, CA, USA) and oxidative stress parameters—superoxide dismutase 2 (SOD2; Cloud-Clone Corp., Houston, TX, USA), catalase (CAT; Cloud-Clone Corp., Houston, TX, USA), reduced glutathione (MyBioSource, San Diego, CA, USA), 4-hydroxynonenal (4-HNE; Biorbyt, Cambridge, UK), malondialdehyde (MDA; Cayman Chemical, Ann Arbor, MI, USA), and advanced glycation end products (AGE; Biorbyt, Cambridge, UK). The assays were performed following the manufacturer's protocols.

Before the determinations, the left ventricle tissue (25 mg) was homogenized in 1 ml of ice-cold phosphate buffer saline (PBS) for measurements of PGE2, PGI2, LTB4, LTC4, LXA4, SOD2, CAT, GSH, 4-HNE, and AGE, or in 250 *μ*l of ice-cold RIPA buffer only for MDA testing. The prepared homogenates suspended in PBS or RIPA buffer were centrifuged as reported by the manufacturer's protocols. After that, the supernatants were transferred into new tubes and frozen immediately at −80°C for analysis.

For the quantitative determinations, the absorbance of all parameters (except for the MDA determination) was detected spectrophotometrically at 450 nm on a microplate reader (Synergy H1 Hybrid Reader; BioTek Instruments, Winooski, VT, USA). The calorimetric measurement at 530 nm was used to assess the content of MDA. The concentration of the analyzed parameters was elaborated depending on the individual standard curves obtained for each measurement. The results are expressed in millimoles (GSH), micromoles (MDA), nanograms (PGI2, LTC4, LXA4, CAT, AGE), or picograms (PGE2, LTB4, SOD2, 4-HNE) per milligram of tissue.

### 2.5. Determination of the Myocardial Anti-Inflammatory and Proinflammatory Cytokines and Chemokines

The left ventricle lysates were prepared in cell lysis buffer (Bio-Rad, Hercules, CA, USA) with the addition of protease inhibitors—factor I and factor II (Bio-Rad, Hercules, CA, USA) and phenylmethylsulfonyl fluoride (PMSF; Sigma Aldrich, Saint Louis, MO, USA). The lysates were centrifuged at 15,000 × *g* for 10 min at 4°C. Subsequently, the obtained supernatants were transferred to new tubes and used to determine the total protein concentration. The range of protein concentrations was 200–900 *μ*g/ml.

The multiplex assay procedure was performed according to the manufacturer's protocol. The concentration of selected cytokines, i.e., granulocyte colony-stimulating factor (G-CSF), granulocyte-macrophage colony-stimulating factor (GM-CSF), growth-regulated oncogenes/keratinocyte chemoattractant (GRO/KC), interleukin 1*α* (IL-1*α*), interleukin 1*β* (IL-1*β*), interleukin 2 (IL-2), interleukin 4 (IL-4), interleukin 5 (IL-5), interleukin 6 (IL-6), interleukin 7 (IL-7), interleukin 10 (IL-10), interleukin 12 p70 (IL-12 p70), interleukin 13 (IL-13), interleukin 17A (IL-17A), interleukin 18 (IL-18), interferon *γ* (IFN-*γ*), macrophage inflammatory protein 1*α* (MIP-1*α*), macrophage inflammatory protein 3*α* (MIP-3*α*), regulated upon activation, normal T-cell expressed and secreted (RANTES), tumor necrosis factor *α*, and vascular endothelial growth factor (VEGF) was measured by the Bio-Plex Immunoassay Kit (Bio-Plex ProRat Cytokine 23-Plex Assay, Bio-Rad; Warsaw, Poland). This procedure is based on multiple assays with covalently coupled magnetic beads. Firstly, a diluted couple of beads were added to each well of the assay plate. After washing the plate twice with Bio-Plex Wash Buffer, blanks, standards, and samples were applied to appropriate wells and incubated for 1 h. Subsequently, the plate was washed, and next, the detection antibodies were added to each well and incubated for 1 h. After adding streptavidin-phycoerythrin (SA-PE) solution, the next incubation, and more washes, the beads were resuspended and added to each well. At last, the 96-well plate was immediately shaken and read on the Bio-Plex 200 System (Bio-Rad Laboratories, Inc.; Hercules, CA, USA) fitted with Bio-Plex Manager Software. The concentration of cytokines was estimated according to the individual standard curves.

### 2.6. Statistical Analysis

All data are expressed as the mean ± standard deviation (SD) based on ten independent determinations in each experimental group, except for the Western blot method, in which results are based on six independent determinations. The statistical assessment was performed using a GraphPad Prism 8.2.1. (GraphPad Software; San Diego, CA, USA). The normality of data distribution and homogeneity of the variance were assessed using the Shapiro–Wilk test and Bartlett's test. The statistical comparisons were performed by a two-way ANOVA followed by a respective post hoc test (Tukey's test and *t*-test). A statistical significance was set at *p* < 0.05.

## 3. Results

### 3.1. Effect of Eight-Week NAC Treatment on the Concentration of Arachidonic Acid in the Plasma of Rats Subjected to a Standard and a High-Fat Diets

**Figure 1:**
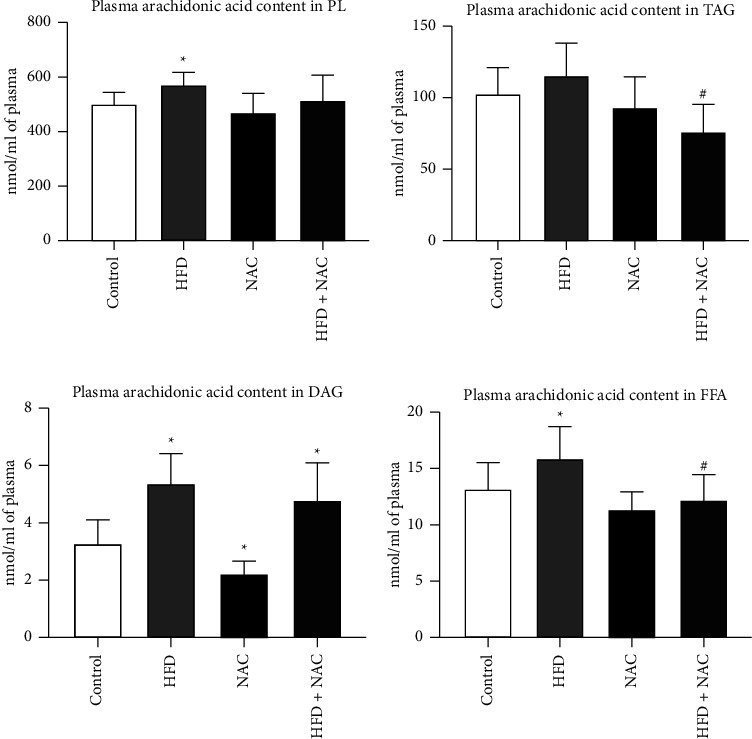
The concentration of arachidonic acid in the (a) phospholipid (PL), (b) triacylglycerol (TAG), (c) diacylglycerol (DAG), and (d) free fatty acid (FFA) fractions after eight-week N-acetylcysteine (NAC) treatment in the plasma of rats fed a standard diet (control) or a high-fat diet (HFD). The arachidonic acid content in the selected lipid fractions was examined by the Gas-Liquid Chromatography (GLC) method. The data are expressed as mean ± standard deviation (SD) and based on ten independent determinations (*n* = 10). The significant differences were assessed by two-way ANOVA followed by a respective post hoc test (Tukey's test and *t*-test). ^*∗*^*p* < 0.05, significant difference compared to the control group; ^#^*p* < 0.05, significant difference compared to the HFD group.

Our study revealed that an HFD caused a significant increase in plasma AA concentration in the PL fraction (HFD: +14.9%, *p*=0.0393, vs. control group; Figure 1(a)). No obvious changes in the phospholipid's AA content were observed in rats treated with NAC (NAC: *p*=0.5320, vs. control group, HFD + NAC: *p*=0.6206 and *p*=0.2595, vs. the control and HFD groups; Figure 1(a)). In the TAG fraction, NAC supplementation to rats fed an HFD resulted in a crucial reduction in the AA level (HFD + NAC: −34.6%, *p*=0.0040; Figure 1(b)) compared to the HFD group, which is the objective manifestation of impaired inflammation development. In relation to the standard condition, there were no significant changes in all examined groups (HFD: *p*=0.4206, NAC: *p*=0.8413, HFD + NAC: *p*=0.0599; Figure 1(b)). Furthermore, in the DAG fraction, the AA content was noticeably increased in the HFD and HFD + NAC groups (HFD: +64.7%, *p*=0.0035, HFD + NAC: +47.3%, *p*=0.0129; Figure 1(c)) and noticeably decreased in the NAC alone group (NAC: −33.7%, *p*=0.0002; Figure 1(c)) in relation to the control group. Plasma AA levels in the DAG pool did not substantially differ in the HFD with NAC treatment group (HFD + NAC: *p*=0.2014; Figure 1(c)) compared to the HFD group. As excepted, the considerable increase in the content of AA in the FFA fraction caused by high-fat feeding (HFD: +21.0%, *p*=0.0186, vs. control group; Figure 1(d)) was abolished by the NAC treatment (HFD + NAC: −23.4%, *p*=0.0172, vs. HFD group; Figure 1(d)), indicating a preventive action of NAC on the inflammation development. No effect in the FFA's AA level was observed in rats receiving a NAC alone and in combination with HFD (NAC: *p*=0.1986, HFD + NAC: *p*=0.4685; Figure 1(d)) than in the control group.

### 3.2. Effect of Eight-Week NAC Treatment on the Concentration of Arachidonic Acid in the Left Ventricle Tissue of Rats Subjected to a Standard and a High-Fat Diets

**Figure 2:**
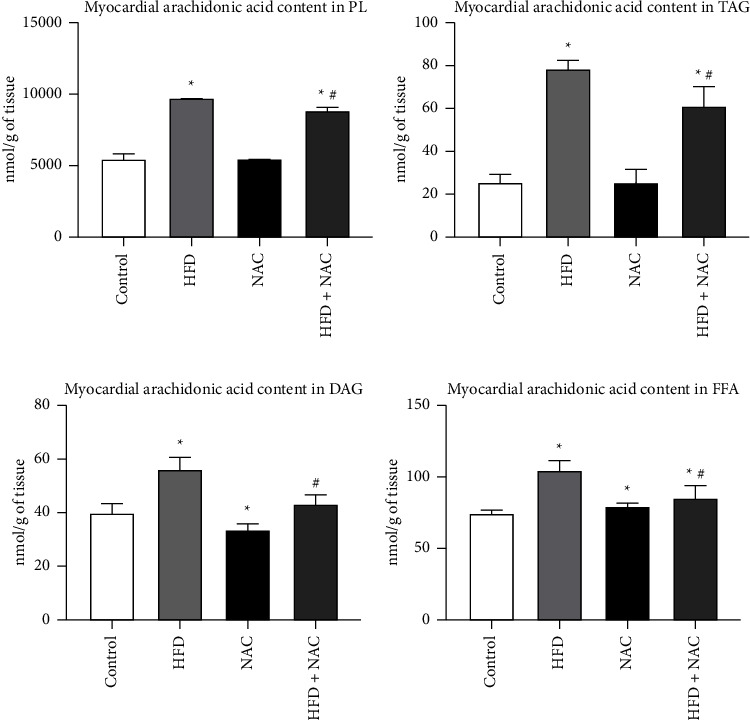
The concentration of arachidonic acid in the (a) phospholipid (PL), (b) triacylglycerol (TAG), (c) diacylglycerol (DAG), and (d) free fatty acid (FFA) fractions after eight-week N-acetylcysteine (NAC) treatment in the left ventricle tissue of rats fed a standard diet (control) or a high-fat diet (HFD). The arachidonic acid content in the selected lipid fractions was examined by the Gas-Liquid Chromatography (GLC) method. The data are expressed as the mean ± standard deviation (SD) and based on ten independent determinations (*n* = 10). The significant differences were assessed by two-way ANOVA followed by a respective post hoc test (Tukey's test and *t*-test). ^*∗*^*p* < 0.05, significant difference compared to the control group; ^#^*p* < 0.05, significant difference compared to the HFD group.

In the lipid overload condition, the arachidonic acid content was considerably elevated in the PL fraction (HFD: +77.6%, *p* < 0.0001, vs. control group; Figure 2(a)). Concomitantly, there were appreciable changes in the myocardial AA content in the PL fraction in rats fed a high-fat diet with NAC supplementation (HFD + NAC: +60.4%, *p* < 0.0001 and −9.7%, *p*=0.0159, vs. control and HFD groups, respectively; Figure 2(a)), which we can suppose that NAC supplementation has a preventive action by the reduction of cardiac AA content in the phospholipid pool. In comparison with the standard condition, we observed a pronounced rise in the arachidonic acid level in the TAG fraction in the HFD and HFD + NAC groups (HFD: +212.6%, *p* < 0.0001, HFD + NAC: +141.2%, *p* < 0.0001; Figure 2(b)). Moreover, NAC administration to rats receiving an HFD caused a significantly diminished the amount of AA in the TAG fraction (HFD + NAC: −22.8%, *p*=0.0079, vs. HFD group; Figure 2(a)) for precautionary the onset of low-grade inflammation. In the NAC alone group we observed no relevant alteration in the AA content in the PL and TAG fractions (PL − NAC: *p*=0.7285; Figure 2(a); TAG − NAC: *p*=0.8853; Figure 2(b)) in relation to the appropriate control group. Our study also revealed that the myocardial AA content in the DAG fraction was remarkably increased in the HFD group (HFD: +41.3%, *p*=0.0007, vs. control group; Figure 2(c)). Additionally, in both NAC-treated groups we noticed a substantial decrease in the concentration of AA in the DAG fraction (NAC: −15.8%, *p*=0.0079, vs. control group, HFD + NAC: −23.2%, *p*=0.0001, vs. HFD group; Figure 2(c)), indicating a prophylactic NAC effect on an early indicator of inflammation. In the HFD + NAC group we noticed no markedly elevation in the diacylglycerol's AA content (HFD + NAC: *p*=0.2222; Figure 2(c)) compared to the standard condition. The content of arachidonic acid in the FFA fraction was substantially enhanced in all examined groups (HFD: +40.2%, *p* < 0.0001, NAC: +6.7%, *p*=0.0218, HFD + NAC: +14.3%, *p*=0.0411; Figure 2(d)) in relation to the control group. We also observed a pronounced decrease in the AA level in the FFA fraction (HFD + NAC: −18.5%, *p*=0.0009; Figure 2(d)) than the HFD group, so we can suppose that NAC has an anti-inflammatory properties.

### 3.3. Effect of Eight-Week NAC Treatment on the Expression of Proteins from Eicosanoid Synthesis Pathway in the Left Ventricle Tissue of Rats Subjected to a Standard and a High-Fat Diets

**Figure 3:**
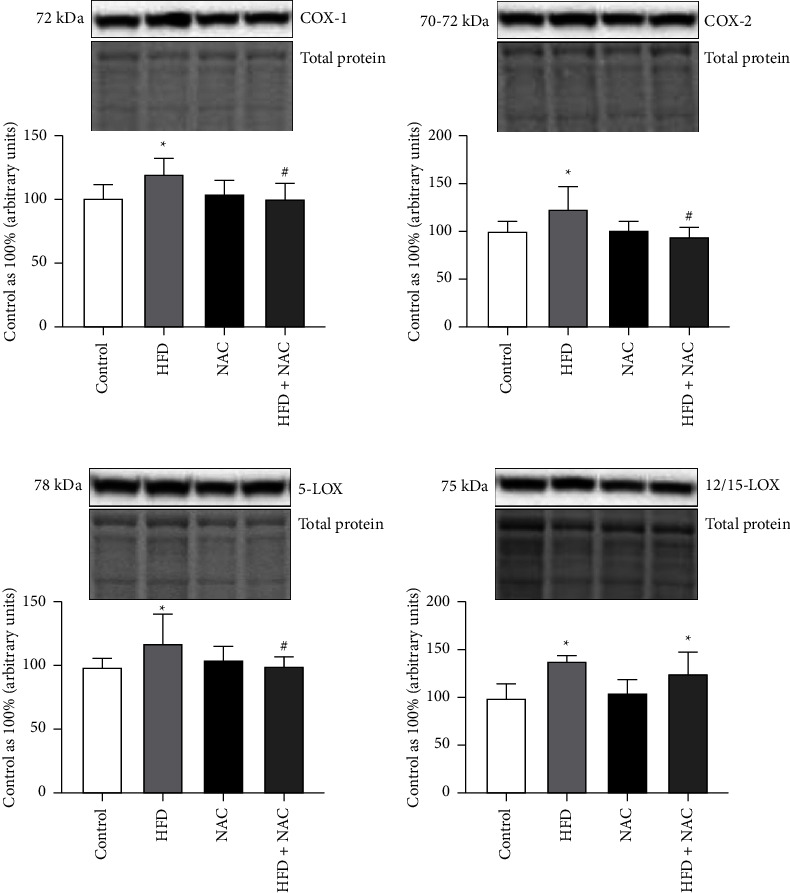
The expression of proteins involved in the eicosanoid synthesis pathway, i.e., (a) cyclooxygenase-1 (COX-1), (b) cyclooxygenase-2 (COX-2), (c) 5-lipoxygenase (5-LOX), and (d) 12/15-lipoxygenase (12/15-LOX) after eight-week N-acetylcysteine (NAC) treatment in the left ventricle tissue of rats fed a standard diet (control) or a high-fat diet (HFD). The expression of selected proteins was examined by the Western blot method. The data are expressed as mean ± standard deviation (SD) and based on six independent determinations (*n* = 6). The significant differences were assessed by two-way ANOVA followed by a respective post hoc test (Tukey's test and *t*-test). ^*∗*^*p* < 0.05, significant difference compared to the control group; ^#^*p* < 0.05, significant difference compared to the HFD group.

Under lipid overload condition, we noticed a significant increment in the total expression of COX-1 (HFD: +20.0%, *p*=0.0022, vs. control group; Figure 3(a)), which was abolished by the chronic NAC administration (HFD + NAC: −16.5%, *p*=0.0046, vs. HFD group; Figure 3(a)), thus revealing its preventive effect on inflammation occurrence. Similar protective influence was disclosed in the COX-2 expression (HFD: +24.4%, *p*=0.0263, vs. Control group, HFD + NAC: −23.9%, *p*=0.0020, vs. HFD group; Figure 3(b)). We also noticed a substantial increase in the expression of 5-LOX in rats fed a high-fat diet (HFD: +17.7%, *p*=0.0252, vs. control group; Figure 3(c)), which was abolished by preventive eight-week N-acetylcysteine treatment (HFD + NAC: −15.1%, *p*=0.0078, vs. HFD group; Figure 3(c)). In comparison with proper control group no significant changes induced by NAC alone and HFD with NAC treatment were found in the expression of COX-1, COX-2 and 5-LOX (COX-1 − NAC: *p*=0.4820, HFD + NAC: *p*=0.8204; Figure 3(a); COX-2 − NAC: *p*=0.8109, HFD + NAC: *p*=0.3036; Figure 3(b); 5-LOX − NAC: *p*=0.1702, HFD + NAC: *p*=0.8458; Figure 3(c)). Moreover, the 12/15-LOX expression was remarkably increased in the HFD and HFD with NAC treatment groups (HFD: +39.3%, *p*=0.0070, HFD + NAC: +25.6%, *p*=0.0499; Figure 3(d)) than in the control group. The changes observed in the NAC and HFD + NAC groups in the 12/15-LOX expression did not reach the significant level (NAC: *p*=0.2601, HFD + NAC: *p*=0.2292; Figure 3(d)).

### 3.4. Effect of Eight-Week NAC Treatment on the Concentration of Arachidonic Acid Derivatives in the Left Ventricle Tissue of Rats Subjected to a Standard and a High-Fat Diets

**Figure 4:**
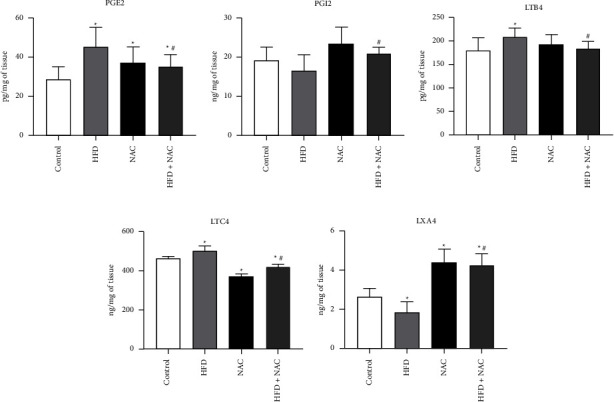
The concentration of arachidonic acid derivatives, i.e., (a) prostaglandin E2 (PGE2), (b) prostaglandin I2 (PGI2), (c) leukotriene B4 (LTB4), (d) leukotriene C4 (LTC4), and (e) lipoxin A4 (LXA4) after eight-week N-acetylcysteine (NAC) treatment in the left ventricle tissue of rats fed a standard diet (control) or a high-fat diet (HFD). The eicosanoids content was examined by the enzyme-linked immunosorbent assay (ELISA) kits. The data are expressed as mean ± standard deviation (SD) and based on ten independent determinations (*n* = 10). The significant differences were assessed by two-way ANOVA followed by a respective post hoc test (Tukey's test and *t*-test). ^*∗*^*p* < 0.05, significant difference compared to the control group; ^#^*p* < 0.05, significant difference compared to the HFD group.

In all examined groups the concentration of PGE2 was significantly elevated (HFD: +57.8%, *p* < 0.0001, NAC: +28.5%, *p*=0.0306, HFD + NAC: +22.7%, *p*=0.0424; Figure 4(a)) in relation to the standard condition. Moreover, NAC supplementation to rats fed an HFD provoked a significant reduction in the PGE2 amount (HFD + NAC: −22.3%, *p*=0.0151; Figure 4(a)) compared to rats receiving a high-fat diet alone. We found that the concentration of PGI2 was no relevant in rats treated by HFD or/with NAC (HFD: *p*=0.2213, NAC: *p*=0.0703, HFD + NAC: *p*=0.2978; Figure 4(b)) in comparison with the control group. In the HFD-induced obesity group, NAC treatment revealed a crucial increment in the PGI2 content (HFD + NAC: +26.0%, *p*=0.0300, vs. HFD group; Figure 4(b)). As excepted, in the HFD group the concentration of LTB4 was greater (HFD: +15.9%, *p*=0.0254; Figure 4(c)) than in the control group. After eight-week NAC supplementation to rats fed an HFD, the concentration of LTB4 was remarkably decreased (HFD + NAC: −11.7%, *p*=0.0070, vs. HFD group; Figure 4(c)). In both NAC-treated group the LTB4 concentration was statistically unchanged (NAC: *p*=0.3017, HFD + NAC: *p*=0.6816, vs. control group; Figure 4(c)). In rats receiving a high-fat diet we noticed a significant increase in the LTC4 content (HFD: +8.2%, *p*=0.0191, vs. control group; Figure 4(d)). Furthermore, in both NAC-treated group LTC4 concentration was relevant impairment (NAC: −20.4%, *p* < 0.0001, vs. Control group, HFD + NAC: −9.4%, *p*=0.0001 and −16.3%, *p* < 0.0001, vs. control and HFD groups, respectively; Figure 4(d)). There was significantly diminished concentration of LXA4 in the HFD group (HFD: −30.7%, *p*=0.0210, vs. control group; Figure 4(e)). In rats treated by NAC application we found a markedly elevation in the level of LXA4 (NAC: +65.0%, *p*=0.0037, vs. control group, HFD + NAC: +59.5%, *p*=0.0007 and +130.0%, *p* < 0.0001, vs. control and HFD groups, respectively; Figure 4(e)). Treatment of NAC implies that obesity-induced inflammation was decreased by the reduction of PGE2, LTB4 and LTC4 levels with simultaneously the elevation of PGI2 and LXA4 levels.

### 3.5. Effect of Eight-Week NAC Treatment on the Expression of Proteins Involved in the Inflammatory Processes in the Left Ventricle Tissue of Rats Subjected to a Standard and a High-Fat Diets

**Figure 5:**
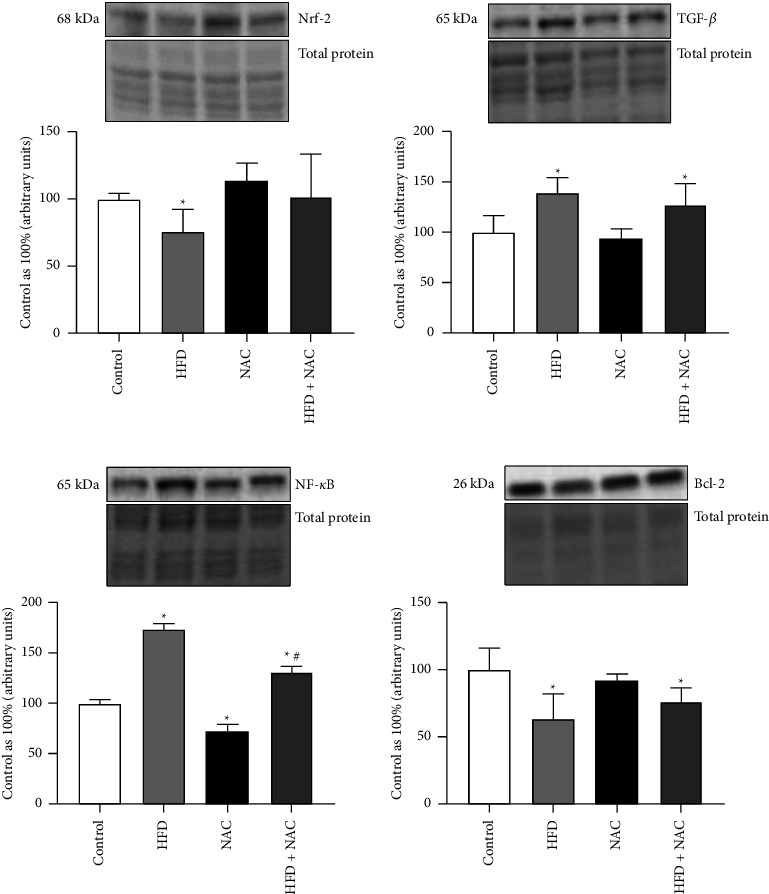
The expression of proteins involved in the inflammatory processes, i.e., (a) nuclear factor erythroid 2-related factor 2 (Nrf-2), (b) transforming growth factor *β* (TGF-*β*), (c) nuclear factor *κ* B (NF-*κ*B), and (d) B cell lymphoma 2 (Bcl-2) after eight-week N-acetylcysteine (NAC) treatment in the left ventricle tissue of rats fed a standard diet (control) or a high-fat diet (HFD). The expression of selected proteins was examined by the western blot method. The data are expressed as mean ± standard deviation (SD) and based on six independent determinations (*n* = 6). The significant differences were assessed by two-way ANOVA followed by a respective post hoc test (Tukey's test and *t*-test). ^*∗*^*p* < 0.05, significant difference compared to the control group; ^#^*p* < 0.05, significant difference compared to the HFD group.

In the left ventricle homogenates, a high-fat diet induced a relevant reduction in the total expression of Nrf-2 (HFD: −24.1%, *p*=0.0035; Figure 5(a)) in relation to the control group. In both NAC-treated groups we observed no significant increase in the Nrf-2 expression (NAC: *p*=0.0617, vs. control group, HFD + NAC: *p*=0.5949 and *p*=0.1121, vs. control and HFD groups, respectively; Figure 5(a)). What is more, in rats fed a high-fat diet alone and a high-fat diet with NAC application the total expression of TGF-*β* was notably increased (HFD: +39.8%, *p*=0.0135, HFD + NAC: +27.2%, *p*=0.0314; Figure 5(b)) compared to the standard condition. In the HFD alone and NAC-treated HFD groups a significant increase in the total expression of NF-*κ*B was noticed (HFD: +73.3%, *p* < 0.0001, HFD + NAC: +30.5%, *p* < 0.0001, vs. Control group; Figure 5(b)). In addition, eight-week NAC treatment disclosed a vital decrease in the expression of NF-*κ*B (NAC: −28.2%, *p*=0.0003, vs. control group, HFD + NAC: −24.7%, *p* < 0.0001, vs. HFD group; Figure 5(c)). The total expression of Bcl-2 was considerably reduced in the rats fed a high-fat diet alone and a high-fat diet with NAC supplementation (HFD: −36.6%, *p*=0.0249, HFD + NAC: −24.0%, *p*=0.0131; Figure 5(d)) in comparison with the control group. The expression of TGF-*β* and Bcl-2 had no significant values in both NAC-treated groups (TGF-*β*–NAC: *p*=0.4736, HFD + NAC: *p*=0.3765; vs. control and HFD groups, respectively; Figure 5(b); Bcl-2 − NAC: *p*=0.4430, HFD + NAC: *p*=0.1722, vs. control and HFD groups, respectively; Figure 5(d)).

### 3.6. Effect of Eight-Week NAC Treatment on the Concentration of Cytokines and Chemokines in the Left Ventricle Tissue of Rats Subjected to a Standard and a High-Fat Diets

A high-fat diet application to rats resulted in a significantly elevated concentration of the following cytokines and chemokines, i.e., G-CSF, IL-1*α*, IL-1*β*, IL-12 p70, MIP-3*α*, RANTES, TNF-*α*, VEGF (HFD: +39.4%, *p*=0.0129; +86.5%, *p*=0.0009; +19.9%, *p*=0.0022; +38.3%, *p*=0.0067; +22.0%, *p*=0.0492; +30.2%, *p*=0.0219; +17.0%, *p*=0.0007; +31.9%, *p*=0.0031; respectively; Table 3) in comparison to the standard condition. Concomitantly, in the untreated HFD group, the concentration of IL-13 was considerably decreased (HFD: −14.4%, *p*=0.0484; Table 3) than in the control group. In addition, eight-week NAC supplementation to rats fed a standard diet caused a substantial decrease in the content of IL-1*α*, IL-1*β*, MIP-1*α*, MIP-3*α*, RANTES, and also TNF-*α* (NAC: −13.3%, *p*=0.0494; −15.2%, *p*=0.0046; −26.1%, *p*=0.0094; −16.1%, *p*=0.0086; −28.6%, *p*=0.0120; −12.8%, *p* < 0.0001; respectively; Table 3) in relation to the control group. Furthermore, we revealed that NAC application to rats fed a high-fat diet caused a significant increase in the concentration of IL-1*α* (HFD + NAC: +18.8%, *p*=0.0167; Table 3) than in the control group. Moreover, in the NAC-treated HFD group, we observed a vital diminishment in the content of selected cytokines and chemokines, i.e., G-CSF, IL-1*α*, IL-12 p70, MIP-3*α*, RANTES, TNF-*α* and VEGF (−26.7%, *p*=0.0188; −36.3%, *p*=0.0022; −27.8%, *p*=0.0019; −31.2%, *p*=0.0115; −22.1%, *p*=0.0045; −12.3%, *p*=0.0067; −21.8%, *p*=0.0024; respectively; Table 3) compared to the HFD alone group, indicating a protective influence of NAC administration on secretion proinflammatory cytokines. In other no listed parameters no statistically significant changes were observed (*p* > 0.05) in all examined group.

### 3.7. Effect of Eight-Week NAC Treatment on the Concentration of Oxidative Stress Parameters in the Left Ventricle Tissue of Rats Subjected to a Standard and a High-Fat Diets

**Figure 6:**
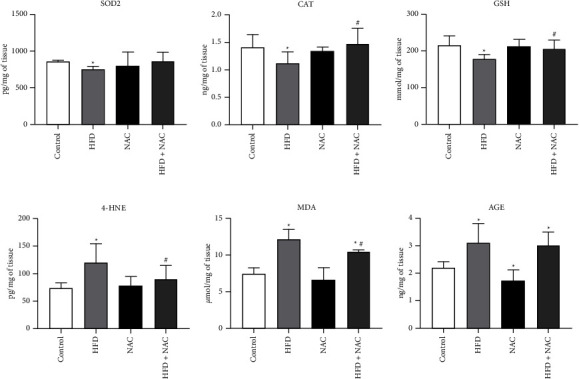
The concentration of antioxidative and oxidative parameters, i.e., (a) superoxide dismutase 2 (SOD2), (b) catalase (CAT), (c) reduced glutathione (GSH), (d) 4-hydroxynonenal (4-HNE), (e) malondialdehyde (MDA), and (f) advanced glycation end products (AGE) after eight-week N-acetylcysteine (NAC) treatment in the left ventricle tissue of rats fed a standard diet (control) or a high-fat diet (HFD). The content of oxidative balance parameters was examined by the enzyme-linked immunosorbent assay (ELISA) and the colorimetric kits. The data are expressed as mean ± standard deviation (SD) and based on ten independent determinations (*n* = 10). The significant differences were assessed by two-way ANOVA followed by a respective post hoc test (Tukey's test and *t*-test). ^*∗*^*p* < 0.05, significant difference compared to the control group; ^#^*p* < 0.05, significant difference compared to the HFD group.

In the HFD alone group the concentration of SOD2 was substantially downgraded (HFD: −12.1%, *p* < 0.0001; Figure 6(a)) than in the control group. Whereas, in the HFD + NAC group the SOD2 level was no statistically different (HFD + NAC: *p*=0.9361 and *p*=0.0627, vs. Control and HFD groups, respectively; Figure 6(a)). Interestingly, in the HFD alone group we noticed a significant decrease in the level of CAT and GSH (CAT–HFD: −20.4%, *p*=0.0293, vs. control group; Figure 6(b); GSH–HFD: −17.1%, *p*=0.0035, vs. Control group; Figure 6(b)), which was restored by the chronic NAC administration (CAT − HFD + NAC: +31.1%, *p*=0.0022, vs. Control group; Figure 6(b); GSH–HFD + NAC: +15.1%, *p*=0.0031, vs. Control group; Figure 6(b)). The preventive effect of NAC was disclosed as the increment in antioxidant capacity, i.e., increases in the content of CAT and GSH levels. The lipid overload conditions disclosed a notable elevation in the concentration of 4-HNE (HFD: +61.9%, *p*=0.0020, vs. control group; Figure 6(d)) whereby this change was abolished by NAC treatment (HFD + NAC: −25.3%, *p*=0.0127, vs. HFD group; Figure 6(d)). Moreover, we found a pronounced increase in the amount of MDA in the HFD and HFD with NAC administration groups (HFD: +63.0%, *p*=0.0001, HFD + NAC: +40.4%, *p*=0.0022; Figure 6(e)) in comparison with the control subjects. In relation to the HFD group, the MDA concentration was remarkedly lower in rats fed a high-fat chow with NAC supplementation (HFD + NAC: −13.9%, *p*=0.0078; Figure 6(e)). The reduction of 4-HNE and MDA levels after application of NAC with HFD may be reflect the protective effect on the deterioration of heart function caused by obesity. In all examined groups the concentration of AGE was appreciable changed (HFD: +41.3%, *p*=0.0022, NAC: −22.0%, *p*=0.0293, HFD + NAC: +37.2%, *p* < 0.0001; Figure 6(e)) than in the control group. In the NAC group the content of SOD2, CAT, GSH, 4-HNE and MDA were statistically unchanged (SOD2 − NAC: *p* > 0.9999; Figure 6(a); CAT − NAC: *p* > 0.9999; Figure 6(b); GSH − NAC: *p*=0.8290; Figure 6(c); 4-HNE − NAC: *p*=0.6753; Figure 6(d); MDA − NAC: *p*=0.1620; Figure 6(e)) than in the proper control group. Moreover, in rats receiving an HFD with NAC solution no obvious changes in the 4-HNE and AGE levels were observed (4-HNE − HFD + NAC: *p*=0.1128, vs. control group; Figure 6(d); AGE − HFD + NAC: *p* > 0.9999, vs. HFD group; Figure 6(f)).

## 4. Discussion

Inflammation is a concomitant factor in obesity-related cardiovascular diseases, contributing to the progression of myocardial fibrosis and loss of heart function [19]. The regulation of the inflammatory response is based on the control of the level and biological activity of mediators, commonly known as eicosanoids. We investigated the effect of N-acetylcysteine on AA derivatives as key parameters of inflammation state and related parameters of oxidative balance in the left ventricle tissue of HFD-fed obese rats (the precise characterization of the obesity model in rats was previously described in our studies [20–22]). In our research, chronic oversupply of FA caused an increase in the myocardial AA content in each examined fraction, i.e., PL, TAG, DAG, and FFA. Similarly, an elevated concentration of AA in plasma PL, DAG, and FFA fractions was observed. The increment of AA content in the various lipid pools suggests the development of inflammation in the heart, which may deteriorate heart functioning [23]. In line with our observations, the results obtained by Pakiet et al. showed an increase in the content of n-6 PUFA, including AA, in the FFA and PL fractions in the plasma as well as in the heart tissue of HFD-treated mice [24]. Interestingly, we exhibited that NAC supplementation ameliorated an increase in myocardial arachidonic acid concentration induced by HFD-feeding in all examined fractions. We can suppose that the present reduction in AA concentration is the result of NAC supplementation revealing its anti-inflammatory properties. The AA pathway remains under the control of two crucial types of enzymes, i.e., cyclooxygenases catalyzed the generation of 2-series prostaglandins and thromboxanes (TX), and also lipoxygenases (LOX), which participate in the formation of 4-series leukotrienes (LT) and lipoxins (LX) [25]. In our research, a high-fat diet resulted in elevated myocardial expression of both COX isoforms (COX-1 and COX-2), which mediates the production of prostanoids. Studies conducted on hypertensive rats revealed that animals receiving an HFD for 10-week had an increased COX-1 expression with no changes in COX-2 expression in the aorta [26]. On the other hand, in research conducted on mice fed an HFD for 8-week a rise in COX-2 expression in the myocardial tissue was reported [27]. This small discrepancy in COX-2 expression may be a result of different experimental materials—aorta and myocardium. COX-1 is constitutively secreted by most mammalian tissues and is called a “housekeeping” enzyme that regulates metabolism processes under physiological conditions [28]. COX-1 is mainly responsible for the production of thromboxane A2 (TXA2), but PGI2 is also responsible for maintaining cell integrity. In addition, PGI2 is considered a potent vasodilator and inhibits platelet aggregation [29, 30]. Many studies have noticed that PGI2 attenuates cardiac hypertrophy and fibrosis through inhibited collagen synthesis [30, 31]. On the other hand, COX-2 is an enzyme, which expression is revealed in response to the development of inflammation state and might be enhanced by the release of proinflammatory cytokines [29]. Moreover, COX-2 regulates the first step of AA conversion to proinflammatory PGE2 and also anti-inflammatory PGI2 which contribute to the inflammatory response [23]. Several findings showed that PGE2 levels increase in cardiovascular diseases, e.g., cardiac hypertrophy or myocardial fibrosis [32, 33]. In our study, we demonstrated that an enhanced expression of COX-2 resulted in a rise in PGE2 level, which was abolished by N-acetylcysteine supplementation. Our observations are consistent with Guo et al.' data in which 8-week NAC administration decreased the expression of COX-2 in the heart of diabetic rats [34]. NAC intensified the influence of common nonsteroidal anti-inflammatory drugs (rofecoxib and diclofenac) by inhibiting COX-2 activity, resulting in a significant reduction in PGE2 level, which was observed by Hoffer et al. on human monocytes with lipopolysaccharide-induced PGE2 formation [2]. The above data show a decline in the content of PGE2 after N-acetylcysteine treatment, thus pointing to a potential role of NAC in inflammation-associated cardiac obesity. As we mentioned, LOX is a second major enzyme family involved in the AA metabolism pathway, leading to the formation of anti-inflammatory LX and proinflammatory LT [23]. In our study, we demonstrated an elevation in the total expression of 5-LOX after 8 weeks of high-fat feeding. As a consequence of the mentioned alteration in 5-LOX expression, the concentration of LTB4 and LTC4 increased. LTB4 is one of the crucial chemoattractant in the early phase of inflammation that causes the infiltration of neutrophils [35]. LTB4 also participates in the development of atherosclerosis and myocardial impairment [29]. In contrast, LTC4 is associated with the occurrence of oxidative stress and apoptosis in the myocardial tissue [36]. Becher et al. revealed that the incubation of cardiomyocytes with LTC4 led to an elevated generation of reactive oxygen species, resulting in the activation of the apoptosis process, which was reflected in fragmented and/or pyknotic cell nuclei. Importantly, pharmacological inhibition of the of LTC4 receptor prevented ROS production and simultaneously attenuated cardiomyocyte apoptosis [36]. The above effects prove that LTB4 and LTC4 weaken myocardial function. In our study, the administration of NAC abolished the increased 5-LOX expression, which resulted in a decline in the content of LTB4 and LTC4. Following our results, the study conducted by Karuppagounder et al. disclosed that NAC prevented against hemin-induced ferroptosis through scavenger proinflammatory lipid derivatives such as LTB4 and LTC4 generated by 5-LOX activity in nerve cells [37]. We can presume that N-acetylcysteine has a protective influence on the inflammation state in HFD-induced cardiac injury by ameliorating the expression of 5-LOX and the generation of 4-series leukotrienes. Herein, we also demonstrated a significant increase in the total expression of 12/15-LOX in cardiac tissue of HFD-treated rats. A previous study showed that 3-month of high-fat feeding prompted a rise in the expression of 12/15-LOX in the arteries of wild-type mice, causing the breakage of tight junctions and macrophage adhesion that underlie the mechanism of atherosclerosis [38]. The data also suggested that an enhanced expression of 12/15-LOX in a late inflammation state could result from an increased PGE2 level [23]. It is important to note that 12/15-LOX catalyzes the synthesis of 4-series lipoxins such as LXA4, which concentration in the HFD group was decreased. LXA4 is considered an atheroprotective factor due to the inhibition of proinflammatory cytokines generation and the prevention of neutrophil chemotaxis [23, 29]. In the present study, chronic NAC treatment of rats fed an HFD enhanced the LXA4 level in the left ventricle, which may limit obesity-induced inflammation by decreasing proinflammatory cytokines content [39]. We also demonstrated a decrease in the expression of Nrf-2 and Bcl-2 with simultaneous increases in the expression of NF-*κ*B in the left ventricle of obese rats, which regulates the synthesis of inflammatory cytokines and chemokines [40, 41]. In light of these reports, we observed that in obese rats NAC ameliorated myocardial NF-*κ*B expression, inhibiting the production of proinflammatory cytokines, such as TNF-*α*. Another study conducted on a rabbit model with doxorubicin-induced heart failure showed that NAC, by increasing the total antioxidant capacity and reducing NF-*κ*B activation, diminished cardiomyocyte apoptosis and the expression of proinflammatory 8-iso-prostaglandin F2*α*. This finding suggests that NAC improves the structure and functioning of the myocardium under an inflammation state [42]. So, it may be presumed that in the left ventricle of rats from the HFD + NAC group we observed a decrease in the total expression of NF-*κ*B, which led to a weakness in the inflammation processes by reducing the level of cytokines, i.e., IL-1*α* and TNF-*α*. What is more interesting, we also revealed a significant influence of NAC on inflammatory parameters in the cardiac tissue of rats fed a standard diet. In our research, the chronic administration of NAC to rats from the control group caused the diminishment of AA concentration in the DAG and FFA pools along with a decrease in the myocardial content of its proinflammatory eicosanoid derivatives, i.e., PGE2 and LTC4. According to existing literature, the impact of NAC on inflammatory mediators in an animal model fed a standard diet is unclear. Following our results, studies conducted on astrocytes cultured under normoxic conditions showed that NAC reduced the release of arachidonic acid into the media, which can potentially be responsible for the development of inflammation, and thus protected against the toxic effects of AA [1]. Concomitantly, we also observed the reduction in NF-*κ*B expression in the left ventricle from the control rats treated with NAC. In line with this alteration, a decrease in the content of the following proinflammatory cytokines: IL-1*α*, IL-1*β*, MIP-1*α*, MIP-3*α*, RANTES, TNF-*α* was observed, limiting the occurrence of inflammation. The reduction of the above-mentioned parameters in the group of rats fed a standard chow indicates the beneficial role of NAC as an anti-inflammatory compound preventing the development of heart dysfunction.

It is known that oxidative stress is one of the important factors for cardiac damage incidence. Oxidative stress is defined as an imbalance between the antioxidant capacity and the generation of reactive oxygen species and free radicals [43]. There is some evidence showing a decrease in the content of enzymatic antioxidants—SOD2, CAT, and nonenzymatic antioxidants—GSH in CVD induced by high-fat feeding [44, 45]. In line with the above reports, in our study, the content of antioxidant markers, i.e., SOD2, CAT, and GSH, was decreased during feeding with HFD. It should be noted that reduced glutathione is a common antioxidant in the cardiovascular system that plays a major role in the inactivation of ROS or as a cofactor for glutathione peroxidase, causing the degradation of hydrogen peroxide [46]. Thus, GSH restores intracellular redox balance and also inhibits the inactivation of nitric oxide generated by the endothelium, altering vasomotor reactivity [43]. In agreement with our study, Andrich et al. revealed a decreased GSH level in the skeletal muscle of rats fed an HFD for 2 weeks [47]. In our study, NAC supplementation increased the concentration of GSH in rats fed an HFD. The beneficial effects of NAC administration stem from the counteraction of ROS and the supplementation of a greater content of cysteine, which is a precursor for the most common antioxidant—GSH [46, 48]. De Mattia et al. showed that NAC treatment caused an increase in GSH concentration and ameliorated the adhesion of molecules to the endothelium, ensuring its proper functioning [49]. In turn, catalase also exhibits action against cardiac oxidative injury by catalyzing the decomposition of hydrogen peroxide and inactivating this reactive form of oxygen into nontoxic products [50]. Mabrouki et al. demonstrated a reduction in CAT, and SOD levels in cardiac tissue within 12 weeks of HFD-treated rats [51]. In our study, an 8-week NAC application also restored the lowered level of CAT in rats receiving an HFD. Qin et al. demonstrated that catalase overexpression in a cardiomyopathy-transgenic mice model prevented against cardiac remodeling and its progression to heart failure [52]. In the herein study, we also noticed that feeding an HFD induced an increase in the concentration of lipid glycation and peroxidation products, i.e., 4-HNE, MDA, and AGE in the left ventricle tissue. The research conducted by Hartog et al. revealed that AGE causes the formation of additional bonds called cross-links between extracellular matrix proteins (collagen, elastin, and laminin) decreasing their elasticity and promoting cardiac dysfunction [53]. Previous studies showed that increased oxidative stress results in the accumulation of AGE, which is associated with the development and progression of myocardial failure [54]. In the case of 4-HNE, it is a toxic lipid peroxidation product, which is formed in the reaction of ROS with cellular biomolecules, such as lipids, especially PUFA, leading to oxidative modifications which further result in impairment in cellular activities [55]. Another product of lipid peroxidation processes is MDA. In patients with coronary heart disease, the serum MDA concentration was increased with a concomitant increase in oxygen-free radicals, indicating the proatherogenic property of this oxidative damage marker [56]. In our study, we observed that alterations in the level of lipid peroxidation products, such as 4-HNE and MDA, induced by HFD were abolished by NAC treatment. Interestingly, Arstall et al.'s study established that NAC administration in combination with standard treatment (streptokinase and/or nitroglycerine) in patients with acute myocardial infarction significantly reduced oxidative stress via diminished MDA content and increased GSH concentration in plasma, resulting in improved left ventricle function [11]. These findings imply that N-acetylcysteine protects cardiomyocytes from damage by decreasing lipid peroxidation products and enlarging the level of antioxidants thereby improving the function of cardiac muscle.

In our study, to obtain a homogeneous group, we used only male rats. There is a lot of evidence that gonadal hormones, especially estradiol in females alter lipid metabolism, leading to higher accumulation or conversion of AA from precursors compared to males [57]. Studies conducted by Lyman et al. showed that female rats maintained an increase content of AA in the plasma PL fraction than did males, pointing to the direct influence of estradiol, as the main cause of this difference [58]. There is one limitation to this study, which does not compare both males and females causing impossible an accurate assessment of NAC effects of on HFD-induced obesity depending on gender. What is more, hyperphagia and weight gain are strongly correlated with sex. Studies demonstrated that male rats presented higher caloric intake and mass body gain caused by HFD compared to females. Further, a delay in HFD-induced obesity and the occurrence of metabolic disturbances due to a lower level of hyperphagia and higher energy expenditure in female rats were observed. Interestingly, female rats are more active than males, resulting in lesser weight gain, which could be one of the possible factors for the obesity resistance in females [59].

## 5. Conclusion

**Scheme 1:**
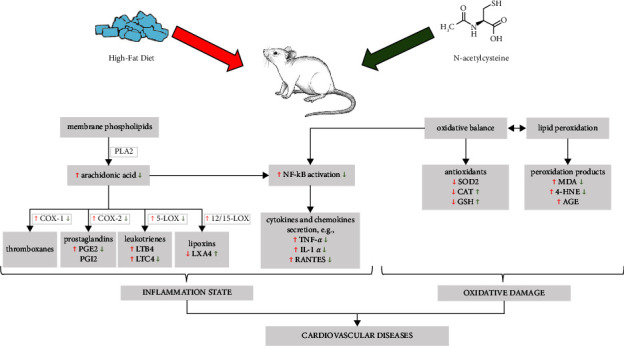
Graphical presentation of the influence of high-fat diet (HFD) alone and combination with N-acetylcysteine (NAC) administration on the inflammation state and oxidative damage in the left ventricle tissue of rats. ↑—increase; ↓—decrease; red arrows show the effect of high-fat diet vs. control group, green arrows show the effect of NAC supplementation to rats fed an HFD vs. HFD group; phospholipase A2 (PLA2), cyclooxygenase-1, -2 (COX-1, COX-2), 5-, 12/15-lipoxygenase (5-LOX, 12/15-LOX), prostaglandin E2 (PGE2), prostaglandin I2 (PGI2), leukotriene B4, C4 (LTB4, LTC4), lipoxin A4 (LXA4), nuclear factor *κ*B (NF-*κ*B), tumor necrosis factor *α* (TNF-*α*), interleukin 1*α* (IL-1*α*), regulated upon activation, normal T-cell expressed and secreted (RANTES), superoxide dismutase 2 (SOD2), catalase (CAT), reduced glutathione (GSH), malondialdehyde (MDA), 4-hydroxynonenal (4-HNE), and advanced glycation end products (AGE).

Obesity is closely related to higher circulating FFA concentrations, which promote ectopic lipid accumulation, thereby adversely affecting cellular structure and functions. These changes lead to the development of inflammation state and oxidative stress, which are important factors for cardiovascular disease occurrence. In this study, we established that NAC supplementation might be a potential agent for preventing the occurrence of inflammation in obesity-related cardiac diseases by a diminishment in the AA concentration, especially in the phospholipid fraction. N-acetylcysteine administration reduced the expression of COX-2, leading to a decrease in the content of proinflammatory PGE2, and also reduced the expression of 5-LOX, resulting in a decrease in the concentration of leukotrienes, namely LTB4 and LTC4. Noteworthy, the observed reduction in the NF-*κ*B expression after NAC supplementation weakened inflammatory signaling via a decline in the content of proinflammatory cytokines. NAC also ameliorated myocardial oxidative stress in rats fed an HFD through an increase in antioxidant content, especially GSH and CAT, with simultaneously a decrease in the level of lipid peroxidation products—4-HNE and MDA. Based on the presented results (Scheme 1) it can be concluded that N-acetylcysteine has a great potential to protect against the development of myocardial inflammation and oxidative stress in rats with obesity induced by a high-fat diet.

## Figures and Tables

**Table 1:** Nutritional composition of the standard rodent diet from Agropol.

Ingredients	Standard diet
Protein, min (%)	23.0
Fat, min (%)	3.0
Ash, max (%)	7.5
Fiber, max (%)	5.0
Lysine, max (%)	1.5
Methionine + cysteine, min (%)	0.8
Calcium, min (%)	1.1
Phosphorus, min (%)	0.7
Sodium, max (%)	0.2
Vitamin A (IU/kg)	8000.0
Vitamin D3 (IU/kg)	1000.0
Vitamin E (mg/kg)	50.0

**Table 2:** Nutritional composition of the high-fat diet (HFD) from Research Diet.

Nutrient classes	Ingredient	High-fat diet (gm %)
Protein	Casein, lactic, 30 mesh	25.8
	Cysteine L	0.4

Carbohydrate	Lodex 10	16.2
	Sucrose, fine granulated	9.4

Fiber	Solka floc, FCC200	6.5

Fat	Lard	31.7
	Soybean oil, USP	3.2

Mineral	S10026B	6.5

Vitamin	Choline bitartrate	0.3
	V10001C	0.1

**Table 3:** The concentration of selected cytokines and chemokines, i.e., granulocyte colony-stimulating factor (G-CSF), granulocyte-macrophage colony-stimulating factor (GM-CSF), growth-regulated oncogenes/keratinocyte chemoattractant (GRO/KC), interferon *γ* (IFN-*γ*), interleukin 1*α* (IL-1*α*), interleukin 1*β* (IL-1*β*), interleukin 2 (IL-2), interleukin 4 (IL-4), interleukin 5 (IL-5), interleukin 6 (IL-6), interleukin 7 (IL-7), interleukin 10 (IL-10), interleukin 12 p70 (IL-12 p70), interleukin 13 (IL-13), interleukin 17A (IL-17A), interleukin 18 (IL-18), monocyte chemoattractant protein 1 (MCP-1), macrophage inflammatory protein 1*α* (MIP-1*α*), macrophage inflammatory protein 3*α* (MIP-3*α*), regulated upon activation, normal *T*-cell expressed and secreted (RANTES), tumor necrosis factor *α* (TNF-*α*), and vascular endothelial growth factor (VEGF) after eight-week N-acetylcysteine (NAC) treatment in the left ventricle tissue of rats fed a standard diet (control) or a high-fat diet (HFD). The cytokines and chemokines content was examined by the bio-plex immunoassay kit. The data are expressed in picograms per milliliter as the mean ± standard deviation (SD) and based on ten independent determinations (*n* = 10). The significant differences were assessed by two-way ANOVA followed by a respective post hoc test (Tukey's test and *t*-test). ^*∗*^*p* < 0.05, significant difference compared to the control group; ^#^*p* < 0.05, significant difference compared to the HFD group.

Parameter	Control	HFD	NAC	HFD + NAC
G-CSF	1.48 ± 0.30	2.06 ± 0.51^*∗*^	1.47 ± 0.25	1.51 ± 0.21^#^
GM-CSF	77.16 ± 8.85	91.14 ± 12.00	76.57 ± 9.07	79.54 ± 13.15
GRO/KC	38.69 ± 0.97	40.17 ± 5.29	37.97 ± 0.78	38.01 ± 0.70
IFN-*γ*	245.84 ± 54.55	295.43 ± 59.06	208.31 ± 25.67	248.83 ± 24.23
IL-1*α*	36.61 ± 2.62	68.29 ± 11.85^*∗*^	31.73 ± 4.31^*∗*^	43.49 ± 4.64^*∗*^^#^
IL-1*β*	35.55 ± 3.81	42.62 ± 5.05^*∗*^	30.16 ± 3.80^*∗*^	38.91 ± 5.16
IL-2	5635.78 ± 136.36	5869.33 ± 401.55	5527.55 ± 823.55	5539.41 ± 544.13
IL-4	3902.38 ± 167.21	3732.38 ± 533.72	4159.25 ± 996.25	3897.48 ± 482.77
IL-5	4125.14 ± 579.13	4121.40 ± 146.46	3730.93 ± 843.59	3805.15 ± 302.33
IL-6	568.41 ± 144.52	625.42 ± 168.93	557.18 ± 81.51	613.76 ± 72.38
IL-7	96.09 ± 5.00	109.34 ± 10.73	94.34 ± 10.98	97.93 ± 11.78
IL-10	214.77 ± 24.52	215.06 ± 31.27	231.74 ± 11.14	217.82 ± 21.08
IL-12 p70	241.28 ± 42.36	333.49 ± 66.82^*∗*^	224.59 ± 56.84	240.88 ± 61.27^#^
IL-13	380.23 ± 57.83	325.57 ± 20.73^*∗*^	410.46 ± 118.36	342.02 ± 60.35
IL-17A	56.81 ± 4.40	65.00 ± 18.62	56.47 ± 13.79	51.83 ± 17.83
IL-18	4062.26 ± 169.82	4540.48 ± 1366.35	3937.21 ± 275.57	4334.51 ± 1217.75
MCP-1	223.38 ± 29.62	250.54 ± 22.84	211.99 ± 17.75	230.30 ± 5.94
MIP-1*α*	7.26 ± 0.90	8.19 ± 1.79	5.36 ± 0.41^*∗*^	7.48 ± 0.74
MIP-3*α*	7.97 ± 0.37	9.72 ± 1.67^*∗*^	6.68 ± 0.69^*∗*^	6.69 ± 1.72^#^
RANTES	198.92 ± 16.32	259.02 ± 35.55^*∗*^	142.11 ± 32.01^*∗*^	201.88 ± 15.91^#^
TNF-*α*	549.88 ± 32.56	643.07 ± 58.75^*∗*^	479.59 ± 25.10^*∗*^	564.09 ± 58.91^#^
VEGF	114.65 ± 13.80	151.20 ± 24.34^*∗*^	109.62 ± 18.15	118.18 ± 22.71^#^

## Data Availability

The data deposited in a manuscript.

## Abbreviations

4-HNE: 4-hydroxynonenal
5-, 12/15-LOX: 5-, 12/15-lipoxygenase
AA: Arachidonic acid
AGE: Advanced glycation end products
Bcl-2: B cell lymphoma 2
CAT: Catalase
COX-1, -2: Cyclooxygenase-1, -2
CVD: Cardiovascular diseases
DAG: Diacylglycerols
FA: Fatty acids
FFA: Free fatty acids
G-CSF: Granulocyte colony-stimulating factor
GM-CSF: Granulocyte-macrophage colony-stimulating factor
GRO/KC: Growth-regulated oncogenes/keratinocyte chemoattractant
GSH: Reduced glutathione
HFD: High-fat diet
IFN-*γ*: Interferon *γ*
IL: Interleukin
LTB4: Leukotriene B4
LTC4: Leukotriene C4
LXA4: Lipoxin A4
MDA: Malondialdehyde
MIP-1*α*, -3*α*: Macrophage inflammatory protein 1*α*, 3*α*
NAC: N-acetylcysteine
NF-*κ*B: Nuclear factor *κ *B
Nrf-2: Nuclear factor erythroid 2-related factor 2
PGE2: Prostaglandin E2
PGI2: Prostaglandin I2
PL: Phospholipids
PUFA: Polyunsaturated fatty acids
RANTES: Regulated upon activation, normal T-cell expressed and secreted
RNS: Reactive nitrogen species
ROS: Reactive oxygen species
SOD2: Superoxide dismutase 2
TAG: Triacylglycerols
TGF-*β*: Transforming growth factor *β*
TNF-*α*: Tumor necrosis factor *α*
VEGF: Vascular endothelial growth factor.
